# Electrocardiogram sonification accelerates detection of ST elevation myocardial infarction compared to analysis based solely on visual display: a randomized controlled simulation study with medical students

**DOI:** 10.1186/s12873-025-01466-8

**Published:** 2026-01-07

**Authors:** Jens Tiesmeier, Friederike Tielking, Steffen Grautoff, Jan Persson, Hans H. Diebner, Thomas P. Weber, Thomas Hermann

**Affiliations:** 1 Institute for Anesthesiology, Intensive Care and Emergency Medicine, MKK-Hospital Luebbecke, Ruhr-University of Bochum, Virchowstr. 65, 32312 Luebbecke, Germany; 2 Department of Anesthesiology, Intensive Care, Emergency and Pain Medicine, MKK-Johannes Wesling Hospital Minden, Ruhr-University of Bochum, Hans-Nolte-Str. 1, 32429 Minden, Germany; 3 Emergency Medical Services, District of Herford, Amtshausstr. 3, 32051 Herford, Germany; 4 Emergency Department, Herford Hospital, Ruhr-University of Bochum, Schwarzenmoorstr. 70, 32049 Herford, Germany; 5 Department of Medical Informatics, Biometry, and Epidemiology, Ruhr-University of Bochum, 44780 Bochum, Germany; 6 Department of Anesthesiology and Intensive Care Medicine, St.Josef-Hospital Bochum, Ruhr-University of Bochum, Gudrunstr. 56, 44791 Bochum, Germany; 7 Ambient Intelligence Group, Faculty of Technology, Bielefeld University, 33619 Bielefeld, Germany

**Keywords:** Electrocardiogram, ECG, ST elevation myocardial infarction, STEMI, Sonification, Biosignal monitoring, Acute coronary syndrome

## Abstract

**Purpose:**

A 12 lead electrocardiogram (ECG) is the standard diagnostic method for the detection of an acute coronary syndrome, as it is also used in emergency medical services. A novel sonification method can convert an important part of the ECG signal into an acoustic signal: The ST segment sonification is particularly useful for the detection of transient ST elevations in patients with suspicion of acute coronary syndrome. A quick and accurate detection of transient ECG changes of the ST segment is prerequisite for proper treatment, thus having immediate therapeutic consequences.

**Methods:**

As part of an emergency training program, a cohort of $n = 44$ medical students was recruited to participate in a two-part study. Some of them, namely $n=32$ of the total 44 subjects, participated in a second part of the study, an RCT, which we report on here. The diagnostic accuracy recently estimated in a classification study involving all 44 subjects with regard to acoustically presented ECG sequences of varying degrees of severity of ST-elevation myocardial infarction forms the background for the RCT described here. The $n=32$ subjects who participated in the RCT were randomly assigned in two-person teams to either an intervention ($n=8$ teams of two) or a control ($n=8$ teams of two) arm, respectively, whereby all teams, except for one dropout due to a technical failure in the intervention arm, went through an emergency simulation where they had to detect an emerging ST elevation myocardial infarction. The intervention group was endowed with a sonification-assisted equipment whereas the control group used standard visual-based ECG diagnosis only.

**Results:**

An adjusted multivariable regression yielded a statistically significant reduction for the intervention group of the delay time from starting a first ECG to the correct diagnosis by 163 seconds ($p = 0.002$) corresponding to $56\%$ of the average delay time in the control group. A subgroup analysis within the intervention arm revealed a notable impact of the attitude toward sonification on delay time between the second ECG and diagnosis. Specifically, increasing disagreement with statement “I conceived the sound of the sonification as pleasant” counterintuitively reduced the delay, whereas an increasing disagreement with “sonification was helpful in making the diagnosis” increased the delay.

**Conclusion:**

The sonification of ECG proved to be significantly superior as an accompanying diagnostic measure in emergency medical services in cases of suspected acute coronary syndrome in a simulated emergency scenario in terms of a proof-of-concept. The established dependence on individual attitudes towards sonification serves to further optimize sonification aesthetics and implementation with a focus on greater alertness and the reduction of stress-induced destraction or alarm fatigue.

**Supplementary information:**

The online version contains supplementary material available at 10.1186/s12873-025-01466-8.

## Introduction

Acute chest pain with the suspicion of acute coronary syndrome (ACS) is a common reason for alerting emergency medical services (EMS) with an incidence of 90–312 per 100,000 inhabitants per year in Europe [1, 2]. The electrocardiogram (ECG) is the diagnostic cornerstone of ACS, especially for the detection of an ST elevation myocardial infarction (STEMI) with an incidence ranging from 44 to 142 per 100,000 inhabitants per year [1, 3, 4].

According to [5], the classical entities of ACS are well known as unstable angina, STEMI, and NSTEMI. However, these entities are not static. The pathophysiology of an ACS after a rupture or an erosion of an unstable coronary artery plaque can result in a dynamic clinical presentation between the aforementioned entities [5]. Typical ECG presentation of NSTEMI are ST segment depression or T-wave inversion assuming a partial vessel occlusion in contrast to ST segment elevation for complete vessel occlusion (STEMI). Therefore, patients with suspected ACS can be a challenge for EMS. According to the current guidelines of the American Heart Association [5] “In patients with suspected ACS in which the intitial ECG is nondiagnostic of STEMI, serial ECGs to detect potential ischemic changes should be performed […]”. In addition, patients with suspected STEMI should be immediately transported to a hospital capable for primary catheterization by the EMS. Sonification, i.e. in the presented context an auditory representation of the ECG signal, has the potential to play an important role as a new supporting tool [6, 7] for a serial and continuously running ECG surveillance without interrupting the patient care and the transport to the destination hospital. In addition, it has recently been reported on the high accuracy of the sonification-assisted classification of different levels of ST elevations and the accurate discrimination from Non-ST elevation (NSTEMI) [8].

The STEMI/NSTEMI paradigm is based on the idea that significant ST elevation on the ECG always indicates acute coronary occlusion. However, this is unfortunately not always the case. On the one hand, ST elevation can occur in the absence of acute coronary occlusion. These ECG patterns are known as “STEMI mimics” [9]. Conversely, it is known that 25% of ACS cases primarily classified as NSTEMI nevertheless turn out to be complete coronary occlusion and are not reclassified as STEMI [10]. Currently, there is a scientific discussion about whether the STEMI/NSTEMI paradigm should be abandoned, because patients with other ECG changes that do not meet STEMI criteria should be treated as urgently as STEMI patients. Some of these ECG changes are already mentioned in current guidelines, e.g. in the ESC Guidelines for ACS [3, 4]. Therefore, some experts suggest changing the distinction between STEMI/NSTEMI to occlusive myocardial infarction and non-occlusive myocardial infarction (OMI/NOMI) [10–14]. This involves considering different ECG patterns in addition to ST elevation. Along these lines, we propose sonification of the ECG as an effective supporting tool for recognition of these ECG patterns.

Sonification is the reproducible, systematic transformation of data to sound and can be used for process monitoring, data analysis or conveying information [15]. Thereby, the sonification of biosignals is a sub-area with applications for supporting diagnostics, particularly the rapid detection of transients [16–19]. In our context, the ECG is used to be transformed into sounds [6, 20, 21]. Well-known examples of biosignal sonifications include the oxygen saturation signal measured by pulse oximetry and the QRS tone, whose measurement belongs to the standard repertoire of medical methods. In our context, proper auditory representations of ST segments of the ECG can yield a spectrum of sounds that allows an accurate discrimination of isoelectric from elevated ST segments [7]. The sonifications used for this study were developed and optimized to transfer the information contained in the biosignals to the EMS crew or, more generally, to an evaluator team [8] and will be described in Sect. 2.

By default rhythm disturbances need to be monitored continuously by one to three leads in order to treat pathological cardiac arrhythmias immediately upon occurrence. However, changes of the ST segment are not covered by this method, but are important for the detection of STEMI. In cases of ACS, fluctuating symptoms are often characterized by transient ECG changes [19]. For these cases, according to the current ESC guidelines [3, 4], a 12 lead ECG has to be recorded on scene within ten minutes after arrival, but routinely only at one point in time [3, 4], which poses a risk of misinterpreting transient ECGs. Furthermore, the evaluation result of the ST segment is dependent on the patient’s age and sex, particularly with respect to leads V2 and V3, according to the current ESC guidelines [3, 4]. It is therefore important to gain expertise and practical skills through intensive training programs in order to ensure correct and timely diagnoses. Particularly, if an ST elevation or other signs of coronary occlusion are present in the ECG, an immediate revascularization is required. Proper treatment includes prompt delivery of medication on scene and urgent heart catheterization. Therefore, optimally the treating hospital should offer 24/7 acute revascularization therapy.

Encouraged by findings of a feasibility study [7] and evidence for a convincing classification quality by means of sonification [8], the hypothesis for the current study was raised: In the presence of a transient STEMI, sonification-assisted diagnosis, when being compared to the standard diagnosis based on a visual ECG-display, reduces the time to diagnosis and increases diagnostic sensitivity in patients with an initially isoelectric ST segment and spontaneously occurring chest pain.

## Material and methods

### ST elevation sonification of ECG signals

Sonification is a versatile technique to transform measured temporal signals into acoustic signals, adaptable for various objectives such as exploratory data analysis, diagnosis, monitoring changes, or classification. In ECG applications, sonification can enhance the detection of cardiac arrhythmias, heart rate variability, and specific cardiac pathologies, such as ST elevation. To focus on ST elevation, a tailored sonification approach is required. A set of designs was discussed in [8], with one selected for further evaluation, summarized below.

In emergency situations, sonification design must balance highlighting important physiological features and minimizing intrusive noise. Design criteria and validation involved expert reviews by sonification designers, clinical partners, and non-clinical experts. Key criteria for the selection of method and design included comprehensibility, dominant perceptual quality, aesthetics, memorizability, compatibility with environmental sounds, and universality. In result, the QRS tones were augmented by a representation that distributed leads over time, anchoring pitch relative to the QRS tone.

**Fig. 1:**
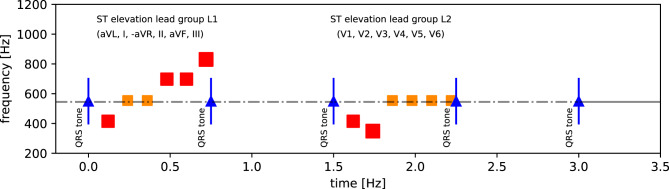
Illustration of the grouped lead scans ST elevation sonification method: lead scans for the Cabrera circle leads and precordial leads are anchored to separate QRS tones, as shown as function of time. Tones’ levels are represented as marker size, sharpness as color (red for sharp, orange for mellow tones), pitch is represented by vertical position, i.e. tones above (resp. below) the dashed line (of the QRS tone frequence) correspond to elevated (resp. suppressed) ST segment

The selected sonification method is an *event-based parameter-mapping sonification*: it extracts as key feature the ST elevation across all 12 ECG leads and maps them onto sound generator parameters to produce structured non-verbal audio signals. These signals accompany the pulse oximeter’s QRS tone, enriching the information with task-specific details. An ST elevation sonification method was conceived, developed, and tested as detailed in a companion paper [8]. In that paper, various designs were discussed, with the “Grouped Lead Scans” design chosen for this study defined as follows (see Fig. 1):

- From the 12 lead ECG, two lists of each 6 voltages derived from the leads are defined. List L1: $\{$aVL, I, $-$aVR, II, aVF, III$\}$, and list L2: $\{V1, V2, V3, V4, V5, V6 \}$. For each lead, the corresponding ST segment value, $st$, is estimated and assigned to one of 5 levels according to the following cutoff rule: (s2) strongly suppressed, i.e. $st \leq -0.2 \mathrm{mV}$, (s1) moderately suppressed, i.e. $-0.2\mathrm{mV} < st \leq -0.1 \mathrm{mV}$, (IE) close to isoelectric with $|st| < 0.1\mathrm{mV}$, (e1) moderately elevated, i.e. $0.2 \mathrm{mV} \geq st \geq 0.1 \mathrm{mV}$, (e2) strongly elevated, i.e. $st > 0.2 \mathrm{mV}$.
- A sequence of short pitched tones (duration $d$, fundamental frequency $f$) of given brightness (number of harmonics $n$) and level ($l$, in dB) is generated for both sets L1 and L2 using an inter-tone interval of $120 \mathrm{ms}$. The tones are pitched relative to the pitch $p_0$ of the QRS tone by ($-8, -5, 0, 4, 7$) semitones corresponding to the 5 cases (s2, s1, IE, e1, e2). The duration $d$ is set to $(100, 80, 50, 80, 100)\,\mathrm{ms}$ according to the 5 cases such that more extreme ST segment values become more salient. Correspondingly, brightness is defined by using $n = (3, 3, 1, 3, 3)$ harmonic overtones, such that ST segments which deviate clearly from IE perceptually stand out. Further, each tone’s level is adjusted by $l = (15, 5, 0, 5, 15) \,\mathrm{dB}$ accordingly. Finally, we use $|st|$ to control the curvature of the corresponding tone’s fade-out envelope such that tones for more extreme $st$ values have a less steep fade out (cf. [8] for details).
- The structured audio message (sequence of 6 tones) for set L1 is triggered by every 8th electrical cardiac activity QRS tone. The sequence for set L2 is triggered two electrical cardiac activities after the L1 sequence, such that both sets of 6 notes can be easily discerned. Listeners thus receive adequately detailed yet aggregated information of the 12 lead ST segment values via two packets of 6 tones, the first for the limb leads along the inverse Cabrera circle with inverted aVR as $-$aVR, the second for the precordial leads from V1 to V6.

For the stimuli used in the study, we used the Laerdal™ Resusci Anne® Advanced SkillTrainer system with a pulse rate of $80$ bpm, and oxygen saturation resulting in QRS tones of fundamental frequency $f_0 = 554$ Hz. Note that the ST-tone’s frequencies $f$ for any given reference frequency $f_0$ and semitone offset $k$ is computed by frequency $f(k) = f_0 2^{k/12}$. Audio examples for several STEMI classes, specifically for an ECG with normal ST segments, anterior STEMI (weak, moderate, severe) and inferior STEMI (weak, moderate, severe) are provided as supplementary material in a previous publication [8], where the results of an expert evaluation process and of a classification study is presented.

### Study protocol

#### Overview

The overall study, the second part of which is discussed here, was designed as a two-part emergency training program, with the first part focusing on the correct classification of different ST elevations based on the acoustically presented ECGs. The second part was designed as a randomized control trial (RCT) in which two groups of students underwent a rescue scenario in a realistic emergency simulation either using the standard diagnostic method (control arm) or with sonification support (intervention arm). The results of the first part of the study, the classification part, which preceded the RCT, have recently been published [8] and provided evidence to justify the RCT. Moreover, since all students participating in the RCT also participated in the preceding classification part, it also served as a preparatory unit and should therefore also be recapitulated here briefly.

As part of the study, basic epidemiological parameters had been collected and questionnaire-based surveys were carried out. A first survey regarding self-ratings based on a 5-point Likert scale of previous experience with emergency situations and musical expertise, as well as questions concerning the general attitude toward sonification took place before the two main parts of the study (classification study and RCT, respectively). A second survey was carried out after the training program (the RCT). Specifically, within the intervention arm only, the second survey addressed the assessment of sonification during the emergency training session (cf. paragraph below for the questionnaires).

#### Participants

After the complete requirements of the ethics committee were met on 23/01/2023, data acquisition began, which ended in 06/2023. The entire study cohort consisted of 44 students from the faculty of medicine of the Ruhr University Bochum (RUB) in Germany (9th and 10th semester) who were assigned to a three day training program in emergency medicine. The emergency simulations took place in groups of two over the study period. All students received a $10$-minute review about diagnosing and treating ACS in pre-hospital care. Since sonification of ST segments is a method previously unknown in practice, the students were given an introduction to this novel methodology, particularly with respect to ECG sonification. To this end, all participants received a $6.25$-minutes introduction video about sonification, its support of ECG monitoring and the motivation of our research. As part of the introduction we presented audio examples of ECGs either in isoelectric mode or with significant elevation of the ST segment to the students. Results of a previous study, which formed the basis for the aforementioned introduction, have recently been published [8].

#### Classification task

Before the simulation study, all 44 students participating in the entire training program took part in a classification study, the results of which have recently been published [8] and are briefly summarized in the following. Regarding the classification of sonified ECG sequences, discrimination of IE (the healthy class) from all other (STEMI) classes yielded both a specificity and a sensitivity of 1 within 660 classification instances. With respect to the exact classification of all 5 classes (IE, inferior/anterior, and moderate/severe STEMI) an overall accuracy of 0.82 (0.79, 0.85) and an intraclass coefficient of $\kappa=0.78$ was estimated. Female students performed significantly better than male. The performance significantly improved in the course of the presentation of three similar classification tasks. This promising result led to raise the hypothesis that sonification is also superior to standard diagnosis in practical EMS operation. As this classification task preceded the RCT, it must also be understood as part of the preparation for the emergency simulation, particularly with respect to the intervention arm. However, structural equality of the two study arms is ensured, as all students completed the classification task.

#### Survey to determine relevant self-assessments and attitudes

At the beginning of the study, questionnaire-based surveys were carried out. The resulting self-assessments and attitudes are partly included as independent variables in regressions to determine the effectiveness of sonification in a realistic emergency setting. On the other hand, they are listed here for the sake of completeness and transparency. A first survey regarding score-based self-assessments of previous experience with emergency situations and musical expertise, as well as questions concerning the general attitude toward sonification took place before the study was carried out. Specifically we asked for subject’s agreement with the following statements on a Likert scale from 1 (not at all) to 5 (completely agree):

- Q1: I had experience with pre-clinical emergencies
- Q2: I have participated to more than 3 emergency trainings
- Q3: I feel confident when handling emergency situations
- Q4: For me, emergency situations cause negative stress

After the perception test, but before the scenario study, which is the focus below, we asked for subject’s agreement based on the same Likert scale as above with the following 3 statements:

- SQ1: Sonification is pleasant to listen to
- SQ2: The sonification is informative, i.e., it enables to identify ST elevation changes in the ECG
- SQ3: I can imagine to listen to these sonifications for a longer time period

#### Simulated medical emergency scenario

The randomized controlled simulation study (sonification as the intervention, visual assessment as control) investigated the use of sonification under more realistic conditions than just context-free classification within the context of an emergency medical scenario with the task to detect an ACS. Thirty-two participants have been randomly assigned by means of a balanced envelope randomization method to either the intervention or the control group such that both study arms each consisted initially of eight teams of two.

When planning the sample size, a realistic expected size of the student cohort of at least $n=40$, i.e. $n=10$ teams of two per study arm, was assumed. Due to the conceptual difference in diagnostic information processing, given the continuous signal transmission, we assumed a huge effect size, however, set a somewhat more moderate value for the power analysis with an effect size of 1.25. Under these conditions, a two-sided t-test with a significance level of 0.05 yields a power of 0.76, which is sufficient for an exploratory study. With the actual number of cases of 8 per study arm, the minimum requirement for an exploratory study was still met, especially since the expected effect size was chosen to be rather moderate.

The task defined for both groups was to record a 12-lead ECG in accordance with the current ESC guidelines, whereby the ECG signals were also presented audibly based on sonification of key properties of ST segments in the intervention group. Initially, the ECG had no evidence of ST elevation. Ninety seconds later the mannequin mentioned additional symptoms (chest pain and dyspnea) which were suggestive to the development of a STEMI and, therefore, should indicate a recording of a second 12-lead ECG. The second ECG presented an anterior STEMI, whereby, as with the first ECG, the second ECG in the intervention group was also presented with sonification.

The primary endpoint was the time gap from the first ECG to the establishment of the correct STEMI diagnosis. The time gap from the second ECG to the establishment of the correct diagnosis served as a secondary endpoint within a subgroup analysis of the intervention arm only. The mutual time delays between the two ECGs, as well as between all major actions (ECG1, ECG2, diagnostic inference, start of treatment, report to rescue control center) carried out serve as secondary endpoints. It should be noted that all outcome variables were determined per team of two, as these variables are based on joint coordinated actions.

Despite the randomized assignment, structural differences in prior knowledge and previous experience can have an impact on the outcome. Therefore, additional relevant parameters were collected via a questionnaire for an adjustment of confounders. Additional questions were asked to determine relevant predictors of performance within the intervention arm. Assessment results on hearing quality, positive benefit, and the impact on the own behavior serve as predictors for a qualitative assessment of the sonification-assisted emergency setting in terms of a subgroup analysis.

#### Medical scenario description

**Fig. 2:**
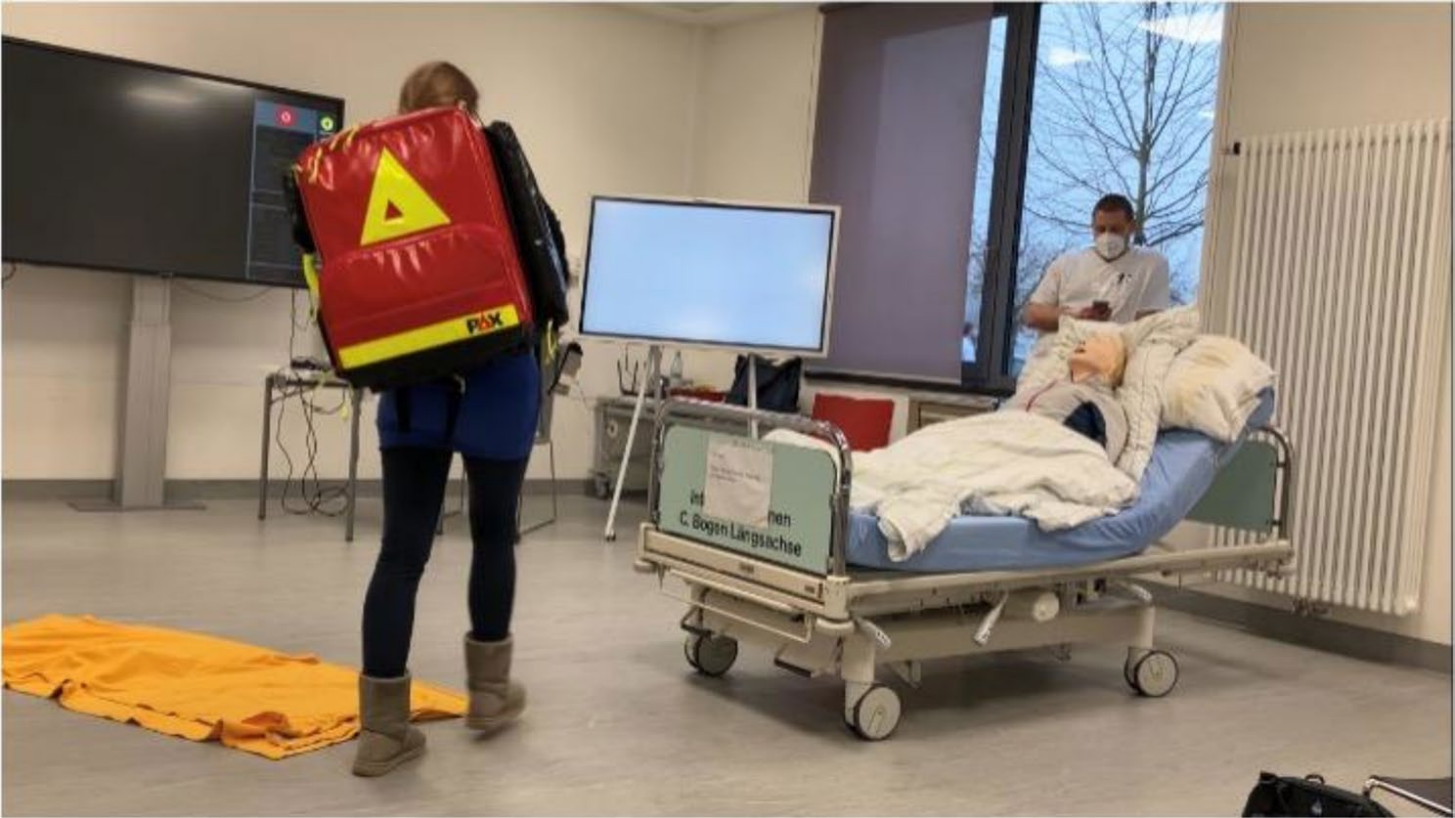
Emergency simulation scenario

The simulated medical scenario depicted in Fig. 2 involved a sixty-six-year-old male patient who called the EMS with left chest pain that had persisted for about an hour and was accompanied by nausea and malaise. In other words, he exhibited a number of symptoms that required attention from the EMS.

As mentioned, each emergency team consisted of two medical students. The patient was represented by the Laerdal™ Resusci Anne® Advanced SkillTrainer. No other persons were actively involved. The information (patient’s voice) was provided by one of the supervising tutors from the “background”. The scenario took place in a training center where a patient’s apartment was simulated. Before the actual scenario started, each team received a brief introduction and was informed about the patient’s symptoms and basic data (name, age, gender). The test subjects had a maximum of 13 minutes to complete the case study.

The emergency equipment consisted of an emergency suitcase, a suction pump, an oxygen bottle and a patient monitor/defibrillator device (Lenovo ThinkPad with Laerdal-Patient-Monitor-Software).

#### Medical assessment

For diagnosis and care, the emergency team should carry out the following measurements and had to perform a series of tasks:

- establishment of basic monitoring (blood pressure measurement, oxygen saturation, 4-lead ECG) for measuring vital parameters
- recording of a 12-lead ECG (initially presented with an isoelectric ST segment)
- recording of a second ECG (now presented with significant elevation of the ST segment 90 seconds after the first ECG, indicative for an anterior STEMI)
- recognizing the ST elevation (myocardial infarction)
- correct selection and application of the indicated drugs
- report to the rescue control center and referring the patient to the nearest appropriate hospital with the option of immediate cardiac catheterization

After establishing the ECG measurement device (i.e. after properly attaching all electrodes) the ECG signal corresponding to a healthy ECG with isoelectric ST segment was visually displayed to all teams, additionally within the intervention arm simultaneously by sonification.

Ninety seconds after the start of the measurement, the ECG turned into an ST elevated ECG signal on the cardiac monitor and, applicable for the intervention group only, the sonification changed into an auditory signal corresponding to the ST elevated ECG. The time interval of approximately ninty seconds was chosen in order to have a defined, comparable setting for all participants on the one hand, and on the other hand because the total duration of the exercise scenario is limited, which necessitated these compromises. Upon request, the patient expressed that the symptoms which initiated the emergency call worsened.

As a consequence of these changes in conditions, the medical team was expected to recognize the ST elevation, correctly select and apply the indicated drugs, pre-announce and admit the patient to an appropriate hospital.

#### Assessment of the scenario by the study participants

After finishing the simulated medical scenario, the participants were asked to complete a second questionnaire. Using a 5-point Likert scale from one (1) corresponding to “strongly agree” through five (5) corresponding to “strongly disagree”, the following statements had to be assessed (FB3 through FB7 intervention group only):

- FB1: I acted confidently during the emergency simulation
- FB2: I felt stressed during the emergency simulation
- FB3: Sonification influenced my individual medical performance
- FB4: Sonification provided a sense of security
- FB5: Sonification was helpful in making the diagnosis
- FB6: I conceived the sound of the sonification as pleasant
- FB7: The use of sonification as a supporting tool in everyday life is conceivable

### Statistical methods

A multivariable linear regression is used to compare the performance of the two study groups (sonification-assisted versus traditional diagnosis as described above). Primary outcome is the time delay from start of the first ECG recording until diagnostic inference. In addition, the delay between the two ECG recordings is conceived as a secondary outcome. Correlations between the delays are reported using Pearson’s coefficients and graphically depicted. In a sub-group analysis applied only to the sonification group, the effect of rating the usefulness of sonification and other self-assessment parameters on the outcome is evaluated.

Univariable 2-sample comparisons are based on t-tests for metric data and Fisher’s exact test or chi-squared test for categorical data. Significance level is chosen to be 5% and precision is reported using 95%-confidence intervals. Within the text, the corresponding confidence interval limits are given comma-separated in brackets after the estimated values. Regression results summarized in tables contain a corresponding column for the confidence intervals.

Statistical analyses and creation of graphs and tables were carried out with statistical programming language R [22].

## Results

### Sonification-assisted ECG surveillance leads to shortened time delay from onset of STEMI to reporting of diagnosis

**Fig. 3:**
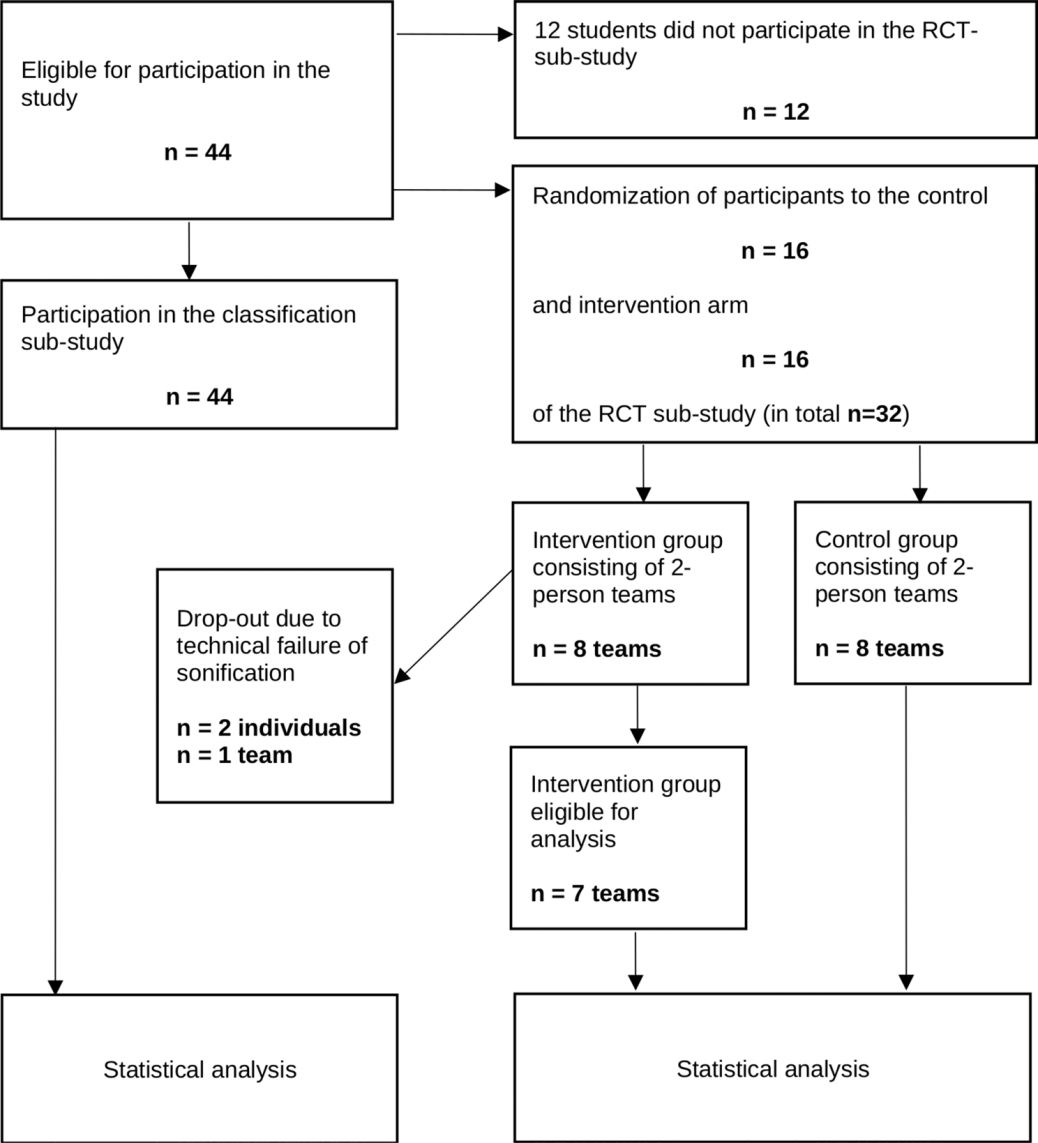
Flow chart of the overall study (classification and training emergency scenario)

This section is devoted to a quantitative analysis of the difference in outcome between the two emergency training groups being compared. The primary outcome measure is the duration from setting up the first ECG to the appropriate STEMI diagnosis by the training participants. One training team that faced a technical error which prevented sonification is excluded from the analysis because an intention-to-treat procedure makes no sense here due to the possible distortion of the result caused by the failure. Thus, 8 teams of two with classic diagnosis (control) and 7 teams of two with sonification-assisted diagnosis (index group) are compared (see Fig. 3).

Table  1 contains summary statistics for demographic and self-assessment parameters of the evaluable RCT participants. Also contained is the date (measured in days from start of the trial) of the training session. Of note, the summary statistics of date have been calculated on the basis of the 15 training teams of two (8 control and 7 index groups), not on an individual basis to avoid irrelevant duplicates. The summary table is stratified by parameter “sonification” indicating the participation in the intervention or in the control group during the training session and does contain the results of univariable tests which confirm the two groups to be structurally similar with the exception of date showing a relevant difference. Please see the Methods Sect. 2 for a detailed description of the covariates.

**Table 1:** Summary statistics of self-assessment scores and demographic parameters stratified by type of training group (sonification used: yes/no) for participants in the emergency training sessions of the RCT. “Q1–Q4” and “SQ1–SQ3” refer to the self-assessment scores collected by means of questionnaires of the same name. “Preknowledge” refers to previous medical training, whereas “musicality” and “instrument” refer to single choice formal information on musical education. FB1 and FB2 are self-assessment scores attributable only to emergency training participants (see the description of the questionnaires used in the Methods section). “Date” refers to the date of the training sessions of the groups of two ($n=8$ for “no” and $n=7$ for “yes”) and is measured in days from study begin

Characteristic	no$^{1}$, *N* = 16	yes$^{1}$, *N* = 14	p-value$^{2}$
Q1	4.00 (3.00, 5.00)	4.50 (4.00, 5.00)	0.5
Q2	4.00 (2.00, 5.00)	4.00 (3.00, 4.00)	0.8
Q3	4.00 (3.00, 4.00)	4.00 (3.00, 4.00)	0.8
Q4	2.50 (2.00, 3.50)	3.00 (2.00, 3.00)	0.9
SQ1	4.00 (2.50, 5.00)	3.00 (3.00, 5.00)	>0.9
SQ2	4.50 (3.00, 5.00)	5.00 (4.00, 5.00)	0.2
SQ3	3.00 (2.00, 5.00)	3.50 (2.00, 5.00)	>0.7
FB1	2.00 (2.00, 3.00)	3.00 (2.00, 3.00)	>0.9
FB2	3.00 (2.00, 3.50)	3.00 (3.00, 4.00)	0.5
Gender			0.4
male	6 / 16 (38%)	3 / 14 (21%)	
female	10 / 16 (63%)	11 / 14 (79%)	
Age	24.0 (23.0, 24.5)	24.0 (23.0, 25.0)	0.6
Musicality	1 / 16 (6.3%)	1 / 14 (7.1%)	>0.9
Instrument	6 / 16 (38%)	9 / 14 (64%)	0.14
Preknowledge	1 / 16 (6.3%)	3 / 14 (21%)	0.3
Date	35 (7, 50)	99 (57, 112)	0.02

^1^ Median (IQR); n / N (%)

^2^ Welch Two Sample t-test; Pearson s Chi-squared test; Fisher s exact test

Table  2 summarizes the outcome variables of the 15 training groups of two, which are the time points of certain actions to be carried out by the students or, where appropriate, the delay times between activities, respectively. Time points of initiation of a first and a second ECG (labelled ECG1 and ECG2) as well as time of diagnosis are reported directly as well as mutual delay times between these events due to the according definitions of the relevant outcome variables. In addition, Table  2 contains the time points of reporting the diagnosis to the rescue control center and the time points of pharmaceutic and non-pharmaceutic interventions initiated by the participants. The treatment activities are mentioned for descriptive reasons but also because they are relevant for future qualitative and quantitative research questions.

**Table 2:** Primary and secondary outcomes (time point of action or time delay between actions) per study group (sonification used: yes/no) measured in seconds. Characteristics “heparin” to “Nitroglycerin” refer to pharmaceutic and non-pharmaceutic interventions. Documented are the time points of the start of intervention measured from the start of the training session. Likewise, “RMLST” and “RMLST2” refer to time of first and second reporting of the diagnosis to the rescue control center. “NA” refers to not applied. The three delay variables refer to time differences as indicated

Characteristic	no$^{1}$ *N* = 8	yes$^{1}$ *N* = 7	p-value$^{2}$
Heparin	472 (402, 557)	441 (336, 467)	0.12
NA	0	2	
Acetylsalicylic acid	460 (402, 542)	410 (291, 429)	0.044
NA	0	1	
Morphine Sulphate	400 (359, 463)	373 (315, 449)	0.6
NA	0	1	
Dimenhydrinate	303 (255, 325)	270 (270, 270)	
NA	5	6	
Oxygen	NA (NA, NA)	267 (216, 345)	
NA	8	3	
Nitroglycerin	430 (335, 525)	407 (375, 446)	0.9
NA	6	3	
ECG1	117 (89, 157)	123 (107, 150)	0.9
ECG2	396 (347, 431)	250 (216, 329)	0.025
NA	1	0	
RMLST	210 (180, 217)	202 (180, 212)	0.3
NA	1	1	
RMLST2	491 (481, 532)	356 (311, 484)	0.006
Diagnosis	450 (379, 500)	260 (220, 356)	0.006
ECG1 to Diagnosis	302 (290, 375)	133 (111, 233)	0.001
ECG1 to ECG2	262 (231, 329)	118 (103, 206)	0.001
NA	1	0	
ECG2 to Diagnosis	36 (28, 58)	10 (4, 27)	0.003
NA	1	0	

^1^ Median (Q1, Q3)

^2^ Welch Two Sample t-testexact test

Although correctly randomized, the average dates of the training sessions (cf. Table  1) differ considerably between the two study arms. Date, therefore, has been considered as confounding variable but did not show a significant effect in an adjusted multivariable regression. However, due to the proper adjustment for date, the effect of the intervention (i.e. sonification) is slightly lowered (main sonification effect $\beta=-163 (-252,-74), p = 0.002$; date effect $\beta=0 (-1,1), p > 0.9$).

Time delay from initiation of the first ECG to diagnostic inference is considered to be one of the primary outcomes of the trial. Table  3 contains the results of a multivariable regression of this outcome on intervention (i.e. sonification), date, and on 8 other independent variables or predictors, i.e. the scores from the survey questionnaires. Although the regression is underpowered according to so many covariates, it still serves to generate hypotheses. Reduced regressions performed as sensitivity analyses can be found in the supplement. Of note, individual scores taken from the questionnaires were summed for the two members of each training group. As the main effect, sonification-assisted diagnosis does have a clinically highly relevant shortening impact on delay time from the start of first ECG to the diagnostic inference, although missing statistical significance ($p = 0.06$) in this underpowered regression model. However, none of the characteristics (gender, preknowledge, attitude toward sonification etc) expressed by the covariates was found to significantly alter the delay. Even the significantly better performance of female participants found in the classification study [8] no longer shows any superiority in this realistic emergency simulation. However, it should be noted that the rescue scenario did not involve differential diagnostic classification of STEMI severity, but rather the time taken to reach the correct decision with only one specified STEMI severity for all teams.

**Table 3:** Result of the linear regression of the delay time from ECG1 to diagnosis on scenario type (with vs w/o sonification), on gender, and on self-assessment scores. Levels of gender refer to the group composition: $0=$both male, $1=$mixed, $2=$both female. Scores FB1 to SQ3 refer to the sum of the scores of the two members per training group. Date has been added for adjustment

Characteristic	Beta	95% CI$^{1}$	p-value
Sonification			
no	$-$	$-$	
yes	−142	−298, 14	0.063
Date	−0.83	−4.6, 2.9	0.5
Gender Composition			
0	$-$	$-$	
1	0.04	−340, 340	0.9
2	2.8	−296, 302	0.9
FB1	−28	−262, 205	0.7
FB2	−3.5	−94, 87	0.9
Q3	14	−114, 143	0.7
Q4	89	−80, 259	0.2
SQ1	−31	−190, 128	0.6
SQ2	21	−105, 146	0.6
SQ3	13	−39, 65	0.5

^1^ CI = Confidence Interval

**Fig. 4:**
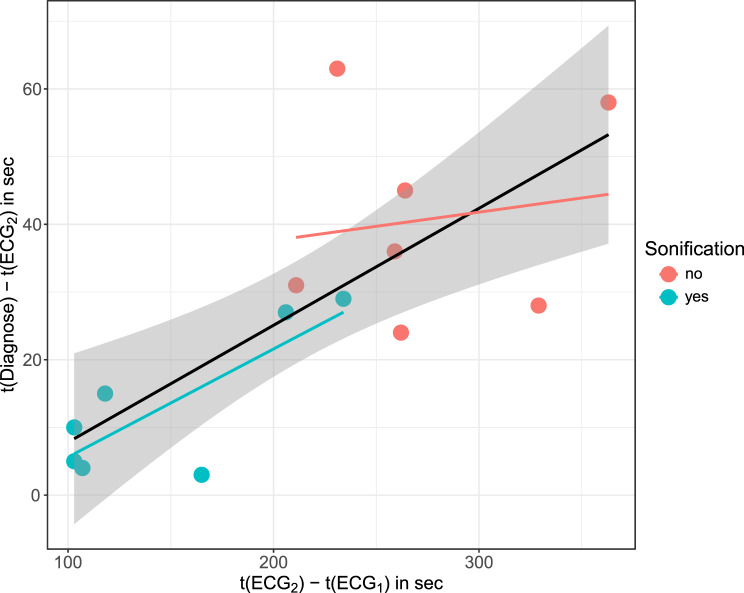
Scatter plot of the second delay (from initiation of second ECG to diagnostic decision, y-axis) versus the first delay (from initiation of first to initiation of second ECG, x-axis). The black line represents the group-independent linear overall regression line (with confidence band), while the group-specific partial regression lines are shown with the corresponding color codes

Regressions of the other two outcomes (delay between ECG1 and ECG2, delay between ECG2 and diagnosis) on the same predictors lead to similar inferences, which is why we refrain from showing the full regression tables. However, one small peculiarity can be observed. As observed with respect to classification (cf. [8]), Q4 actually does unfold a marked impact ($\beta = 11 (-0.02, 22), p = 0.061)$ on the delay time between ECG2 and diagnosis. In line with previous findings [8], we conclude that a general stress-free attitude towards emergency situations correlates with an improved outcome. Finally, the delays from ECG1 to ECG2 and the delays from ECG2 to diagnosis correlate strongly with each other, with the group-independent Pearson correlation coefficient being $r = 0.75$ and the two group-specific correlations being $r = 0.8$ for the sonification group and $r = 0.15$ for the control group. (see Fig. 4).

Thus, it can be concluded that sonification reduces both the delay time from ECG1 to ECG2 and the subsequent duration from ECG2 to STEMI diagnosis. The total duration from ECG1 to diagnosis differs significantly between index and control group. A weak impact of psychological conditions on the duration from ECG2 to diagnosis can be observed. The trial has not been powered to reveal a multiple impact of covariates, therefore, the results point to trends to be validated in larger trials.

### Sub-group analysis indicates a match between perceived and actual benefit of sonification

Only participants in the sonification group have been asked sonification-specific questions FB3–FB7. A regression of the delay time between ECG2 and diagnosis on these 5 scores results in estimates listed in Table  4. Thereby, FB7 was omitted because the corresponding coefficient cannot be estimated due to the low variability of this covariate.

**Table 4:** Results of the subgroup analysis showing the estimates of the regression applied to the index group only. Outcome is the duration from ECG2 to STEMI diagnosis. FB3–FB6 refer to scores derived from questions asked only to the sonification group

Characteristic	Beta	95% CI$^{1}$	p-value
FB3	−13	−41, 14	0.2
FB4	−7.7	−23, 7.3	0.2
FB5	33	11, 54	0.022
FB6	−16	−31, −1.2	0.043

^1^ CI = Confidence Interval

A positive attitude toward sonification (FB5, FB6) does influence delay time, however the effects go in different directions. An increasing disagreement with the statement that “sonification was helpful in making the diagnosis” (FB5) did significantly ($p = 0.022$) increase the delay time, i.e. considerably worsens the performance during the emergency scenario. To the contrary, disagreeing with “I conceive the sound of sonification as pleasant” (FB6) appears to counterintuitively significantly ($p=0.043$) decrease delay time (improves performance).

We refrain from showing the estimates of regressions for the other delay variables since none of them show significant dependence of the outcome on scores FB3 to FB7, however the effects of FB5 and FB6 appear to go into the same directions. We conclude with due caution, that the second delay (between ECG2 and diagnosis) is somewhat increased as a consequence of a pleasant attitude towards sonification which may point to an increased sovereignty that gives rise to a watch-and-wait strategy thus mediated. Of note, there is no effect of date observable within this subgroup analysis. These preliminary findings of this sub-group analysis have to be addressed in suitably designed follow-up studies.

## Discussion

Sonification, i.e. the transformation of data into sound, can serve many different purposes [16]. For example, for blind persons, it opens up opportunities to perceive events that would otherwise remain hidden from them. Here we discussed an application in medical diagnostics that is essentially intended to lower the cognitive load of the medical staff, particularly in stressful situations. For the purposes of a feasibility study, we chose a student training program in the form of a simulated emergency scenario as the study setting. Instead of directly addressing the cognitive load, we followed best practice principles of evidence based medicine and used a patient relevant outcome: delay time from starting the diagnostic procedure to the final establishment of the diagnostic result.

We applied sonification as a monitoring instrument in EMS, specifically to assess the dynamics of ST segment elevations and detect their pathological manifestations. On the one hand, it has been discussed earlier that an auditory display of temporal signals is particularly appropriate to detect dynamically changing patterns in these signals due to the according sensitivity of the ear [16]. On the other hand, the acoustic perception is known to be more strongly coupled to the limbic system when being compared to the visual perception [23], which is why sonification might be conceived as a less objective epistemic instrument. However, this emotional component may even turn into an advantage in emergency situations. Obtaining meaningful information in this respect, ultimately in order to optimize the acoustic design for future applications, was subject of numerous earlier publications (e.g. [16–18, 20]) and this objective was also pursued in the present work. The thus selected sonification method makes stronger ST elevations more salient. It could be argued that greater emphasis is needed for smaller ST elevations, which require more attention to assess the condition, but this would lead to an increase in false negative diagnoses. The proper ?attention-allocation? settings may also depend on user preferences, routine, and staff experience, to name just a few factors, and should be investigated in practical field tests.

Moreover, we addressed emotional and perceptional aspects by conducting accompanying surveys in order to model the resulting scores for various attitudes as influencing factors. The exact significance of the observed dependencies of the scores on the outcomes with respect to the improvement of the sonification design should be determined in follow-up studies, which should also be designed to optimize the emergency training sessions. Thereby, the focus should be on greater alertness and the reduction of stress-induced destraction or alarm fatigue.

To our knowledge, our study is the first RCT to address the superiority of a sonification-assisted diagnosis compared to a visually-based standard diagnosis and to yield a significant result in a largely realistic emergency scenario, even though it was only simulated. No doubt, every second it takes to derive the final STEMI diagnosis within the scope of an EMS counts. The effect is considerably large, such that it gained statistical significance despite adjustments for confounders and a small samples size.

## Limitations

The present study is based on a relatively small sample size. Overall power suffered from the unexpectedly unbalanced temporal allocations to the two study arms. Nevertheless, due to a strong average population effect, it was even possible to adjust for the start time of the training sessions in order to correct a possible bias through different learning gains as a possible confounder, while preserving the significance. Future studies, therefore, should consider stratified randomization.

In order to generate hypotheses, we also performed a multivariable regression analysis by including additional predictors in the model and still achieved a convincing result with regard to the main effect. Given the small sample size, it hardly needs to be emphasized that such a multivariate regression is completely underpowered and can therefore only provide indications as to how a future sufficiently powered study could be designed. Moreover, the multivariable regression tends to overfit due to its low power (cf. the reduced regressions performed as sensitivity analyses in the supplement). Our key statement regarding the significance of the sonification effect, which we were able to demonstrate using the simple regression with only one confounder, namely the date of the respective training session, remains unaffected.

Moreover, the study was not sufficiently powered to obtain significant results with respect to the impact of individual characteristics that may influence the outcome within the sonification group. However, predictors with a relevant influence on the outcome (reduction of the time needed to achieve the diagnosis) were identified, even if these were below the significance threshold. The impact of individual attitudes toward sonification in general and self-confidence in emergency situations, to name but a few, provide very valuable information for improving sonification design parameters and this was indeed one of the intentions of the study presented. However, it must be emphasized once again at this point that the associations identified should by no means be understood in a causal sense. Rather, the results should be used in a hypothesis-generating sense. Experimental study designs that take appropriate strata into account would be desirable in order to obtain more information about the dependencies in question.

Regarding potential learning effects or the effects of relevant preknowledge, in the previously published classification section of the study, which was summarized here in preparation for the RCT, we explicitly observed a learning curve through repeated performance of the classification task. In addition, some of the self-assessments collected on prior knowledge also proved to be predictors of performance. We made a similar observation with regard to the RCT reported here, as there were indications of corresponding influences, but the power was only sufficient to formulate corresponding hypotheses. Further unobserved confounders cannot be ruled out and are even likely. In principle, however, at least the self-assessment scores collected proved to be balanced, and it is unlikely that unobserved confounders caused any serious selection bias. Furthermore, since both groups underwent an introductory training session on sonification, a “knowledge contamination” in the control group cannot be ruled out. However, it seems plausible that such “knowledge contamination” renders the comparative study more conservative. Therefore, whether the control group with background knowledge performs better than a comparison group without this knowledge advantage remains a counterfactual question that is beyond the scope of our study.

The aim of the study design was to enable students to recognize transient STEMI. Therefore, the setting was chosen so that a non-pathological ECG sequence was presented first, followed by a pathological one. A pathological sequence presented from the outset might have been recognized less readily than the transient situation that was actually presented. Any possible overestimation resulting from this must be addressed in further studies, but was not addressed in the present study.

With regard to a possible application in real emergency operation, the most significant limitation of the present study is given by the preliminary, ethically necessary restriction to a simulated emergency situation for medical training. Due to the limited time available for such a training session, the procedure was subject to certain conditions, such as choosing approximately 90 seconds between ECGs, which contributes to the artificiality of the simulation, which must be mentioned here as a limiting factor. Thus, a further study based on a real emergency scenario is strongly recommended.

## Conclusions

To conclude, the shown advantage of sonification-assisted STEMI diagnosis within the scope of a simulated EMS now provides sufficient evidence that justifies a further validation in trained EMS personnel with the long-term goal of an RCT in a real-world settings.

## Electronic supplementary material

Below is the link to the electronic supplementary material.

Supplementary Material 1

## Data Availability

Sonification related data, code snippets, as well as sonification related materials have been published as open access supplementary documents to https://doi.org/10.3390/s25144373.

## Code availability

Sonification related code snippets have been published in [8].

## Declarations

### Materials availability

Sonification related materials have been published as open access supplementary documents to [8].
