# Supplementary material for: Electrocardiogram sonification accelerates detection of ST elevation myocardial infarction compared to analysis based solely on visual display: a randomized controlled simulation study with medical students
Source: BMC Emerg Med. 2026 Jan 7;26:30. doi: 10.1186/s12873-025-01466-8 (PMC12849153; doi:10.1186/s12873-025-01466-8)
Supplement: Supplementary file 1 — Supplementary Material 1 [file 12873_2025_1466_MOESM1_ESM.pdf]

Supplementary Document to: Electrocardiogram sonification accelerates detection of ST elevation myocardial infarction compared to analysis based solely on visual display: a randomized controlled simulation study with medical students

Jens Tiesmeier[1*], Friederike Tielking[2], Steffen Grautoff[3,4], Jan Persson[2], Hans H. Diebner[5*], Thomas P. Weber[6], Thomas Hermann[7]

[1*]Institute for Anesthesiology, Intensive Care and Emergency Medicine, MKK-Hospital Luebbecke, Ruhr-University of Bochum, Virchowstr. 65, Luebbecke, 32312, Germany.

[2]Department of Anesthesiology, Intensive Care, Emergency and Pain Medicine, MKK-Johannes Wesling Hospital Minden, Ruhr-University of Bochum, Hans-Nolte-Str. 1, Minden, 32429, Germany.

[3]Emergency Medical Services, District of Herford, Amtshausstr. 3, Herford, 32051, Germany.

[4]Emergency Department, Herford Hospital, Ruhr-University of Bochum, Schwarzenmoorstr. 70, Herford, 32049, Germany.

[5*]Department of Medical Informatics, Biometry, and Epidemiology, Ruhr-University of Bochum, Bochum, 44780, Germany.

[6]Department of Anesthesiology and Intensive Care Medicine, St.Josef-Hospital Bochum, Ruhr-University of Bochum, Gudrunstr. 56, Bochum, 44791, Germany.

[7]Ambient Intelligence Group, Faculty of Technology, Bielefeld University, Bielefeld, 33619, Germany.

*Corresponding author(s). E-mail(s):
Jens.Tiesmeier@muehlenkreiskliniken.de;
hans.diebner@ruhr-uni-bochum.de;
Contributing authors: friederike.brandt@muehlenkreiskliniken.de;
steffen.grautoff@klinikum-herford.de; jan.persson@ruhr-uni-bochum.de;
thomas.weber@kklbo.de; thermann@techfak.uni-bielefeld.de;

Sensitivity analyses

The results of the sensitivity analyses presented in Tables 1 and 2 can be summarized as follows: the full model presented in Table 3 in the main text does not lead to improved goodness-of-fit with respect to the reduced regressions. The reduced regression models are described in the corresponding table captions below. The LRTs from the comparison of the reduced and full models remain insignificant. It follows that the estimated influence of the predictors must be validated in a significantly more comprehensive study and should only be interpreted here as an initial indication. See also the explanations in the Limitations section.

Table 1 Reduced multivariable regression with respect to the full regression shown in Table 3 in the main text. Variable names are the same as in Table 3 of the main text. The three scores SQ1-SQ3 are summed up to a single score SQ. Thereby, score Q4 becomes significant. However, a likelihood-ratio-test with respect to the full model proves to be insignificant with $p = 0.77$. If SQ1-SQ3 are omitted entirely, the p-values of the main effect and of Q4 become slightly smaller, but the LRT remains insignificant.

**Characteristic**	**Beta**	**95% CI**	**p-value**
Sonification			
no			
yes	-146	-228, -64	0.004
Date	-0.39	-1.6, 0.87	0.5
Gender Composition			
0			
1	57	-50, 163	0.2
2	55	-41, 152	0.2
Q3	-2.8	-37, 31	0.9
Q4	59	12, 105	0.020
SQ	0.97	-5.5, 7.4	0.7

CI = Confidence Interval

Table 2 Reduced multivariable regression with respect to the full regression shown in Table 3 in the main text. Variable names are the same as in Table 3 of the main text. Scores SQ1-SQ3 and Gender Composition have been omitted. A likelihood-ratio-test with respect to the full model proves to be insignificant with $p = 0.84$.

**Characteristic**	**Beta**	**95% CI**	**p-value**
Sonification			
no			
yes	-149	-220, -78	<0.001
Date	-0.15	-0.96, 0.65	0.7
Q3	5.0	-24, 33	0.7
Q4	60	18, 101	0.009

CI = Confidence Interval
